# Effect of converting from propofol to remimazolam with flumazenil reversal on recovery from anesthesia in outpatients with mental disabilities: a randomized controlled trial

**DOI:** 10.1186/s12871-024-02526-5

**Published:** 2024-04-22

**Authors:** Sooyoung Jeon, Jieun Kim, Myong-Hwan Karm, Jin-Tae Kim

**Affiliations:** 1 National Dental Care Center for Persons with Special Needs, Seoul National University Dental Hospital, Seoul, Republic of Korea; 2 Department of Dental Anesthesiology, School of Dentistry and Dental Research Institute, Seoul National University, Seoul, Republic of Korea; 3 Department of Anesthesiology and Pain Medicine, Seoul National University Hospital, Seoul National University College of Medicine, 101 Daehak-ro, Jongno-gu, Seoul, Republic of Korea

**Keywords:** Flumazenil, Outpatient anesthesia, Patients with mental disabilities, Recovery of anesthesia, Remimazolam

## Abstract

**Background:**

General anesthesia is often necessary for dental treatment of outpatients with mental disabilities. Rapid recovery and effective management of postoperative nausea and vomiting (PONV) are critical for outpatients. This study aimed to investigate the effect of transitioning from propofol to remimazolam with flumazenil reversal administered toward the end of surgery during propofol-based total intravenous anesthesia (TIVA) on recovery.

**Methods:**

Adults with mental disabilities scheduled to undergo dental treatment were randomly assigned to receive either propofol-based TIVA (Group P) or propofol-remimazolam-based TIVA with flumazenil reversal (Group PR). Propofol was replaced with remimazolam 1 h before the end of surgery in Group PR; moreover, 0.5 mg of flumazenil was administered after the neuromuscular blockade reversal agent. The primary outcome was the duration of stay in the post-anesthesia care unit (PACU). The secondary outcomes included time to eye-opening, time to extubation, occurrence of PONV, and quality of recovery.

**Results:**

Fifty-four patients were included in this study. The duration of stay in the PACU in Group PR was significantly shorter than that in Group P (mean difference, 8.7 min; confidence interval [95% CI], 3.3–14.2; *P* = 0.002). Group PR exhibited a shorter time to eye opening (mean difference, 5.4 min; 95% CI, 3.3–8.1; *P* < 0.001) and time to extubation (mean difference, 5.5 min; 95% CI, 3.6–7.9; *P* < 0.001) than Group P. Neither group required the administration of rescue analgesics, and the incidence of PONV was not reported.

**Conclusions:**

Transitioning from propofol to remimazolam 1 h before the end of surgery followed by flumazenil reversal reduced the duration of stay in the PACU and the time to eye opening and extubation without affecting the incidence of PONV and quality of recovery.

**Trial registration number:**

Clinical Research Information Service (KCT0007794), Clinical trial first registration date: 12/10/2022.

## Background

General anesthesia is often the only feasible option for patients with mental disabilities who have difficulty cooperating during dental treatment [1, 2]. Since most dental treatments are typically conducted on an outpatient basis, it is crucial to ensure prompt recovery and effectively address postoperative nausea and vomiting (PONV) [2, 3]. However, patients with mental disabilities tend to awaken slowly from general anesthesia compared with those without mental disabilities. Moreover, this effect is exaggerated among patients receiving antiepileptic medications [4, 5].

Propofol is a short-acting intravenous anesthetic characterized by rapid anesthesia induction and recovery as well as a reduced incidence of PONV and postoperative pain, which makes it an appropriate anesthetic agent for outpatient settings [6–8]. However, it has no reversal agent, and its propofol infusion time is positively correlated with the context-sensitive half-time. This can result in a substantial delay in recovery and may even lead to respiratory depression in vulnerable patients [8–10].

Remimazolam is a benzodiazepine with ultrashort-acting properties, characterized by rapid onset and offset [11, 12]. It has a short context-sensitive half-time owing to its rapid plasma clearance mediated by nonspecific esterase, even when administered over extended periods [13]. Furthermore, its action can be reversed by flumazenil, and it has demonstrated hemodynamic stability in numerous clinical trials [14–17]. Specifically, it has demonstrated hemodynamic stability in clinically vulnerable patients, especially those classified as American Society of Anesthesiologists (ASA) physical status class III [18]. However, despite these advantages, some studies have reported delayed recovery of consciousness from remimazolam-based total intravenous anesthesia (TIVA) without flumazenil reversal compared with that from propofol-based TIVA [14]. Several randomized clinical trials have demonstrated that the additional use of flumazenil for reversal in remimazolam-based TIVA facilitates faster recovery [15, 17, 19] and a shorter stay in the post-anesthesia care unit (PACU) than those of propofol-based TIVA [19].

Nevertheless, remimazolam-based TIVA is associated with a longer time to loss of consciousness than propofol-based TIVA [14, 16, 19], which can impede anesthesia induction in uncooperative patients with disabilities who require rapid induction. In addition, remimazolam-based TIVA has a higher incidence of PONV than propofol-based TIVA [14, 20]. Furthermore, remimazolam is relatively expensive; thus, using remimazolam for the entire procedural time can lead to higher out-of-pocket expenses for patients than using propofol.

We hypothesized that by combining the rapid onset and low incidence of PONV associated with propofol with the swift recovery facilitated by flumazenil reversal of remimazolam-based anesthesia, we could administer more suitable anesthesia to outpatients with mental disabilities. Thus, this prospective, randomized controlled trial aimed to evaluate whether replacing propofol with remimazolam 1 h before the completion of dental treatment, followed by reversal with flumazenil, enhances postoperative recovery in patients undergoing dental procedures. Moreover, our study aimed to compare postoperative outcomes between propofol-based TIVA and propofol-remimazolam-based TIVA with flumazenil reversal.

## Methods

### Study design

This prospective, parallel-designed, single-center, randomized, single-blind, controlled study was conducted at the National Dental Care Center for Persons with Special Needs at Seoul National University Dental Hospital. The study protocol was approved by the Institutional Review Board of the Seoul National University Dental Hospital (IRB # CME22002; date of approval: 11/8/2022) and registered with the Clinical Research Information Service (number: KCT0007794; date of registration: 12/10/2022). This study adhered to the principles outlined in the Declaration of Helsinki, and written informed consent was obtained from the parents or legally authorized representatives of the patients before enrollment.

This study included mentally disabled adults with ASA physical status II–III scheduled to undergo dental treatment under general anesthesia. A mental disability is defined as a cognitive or psychological condition that limits significant life activities or require special care; such conditions include intellectual disabilities, developmental disabilities, autism, or dementia. The exclusion criteria were as follows: a history of an allergic reaction to any study medication; body mass index (BMI) ≥ 35 kg m^− 2^; an expected surgery duration of ≤ 1 h; previous participation in a study; and having undergone a tracheostomy. Further, we excluded patients who had received anxiolytics, hypnotics, or antipsychotics within 24 h before the administration of general anesthetics, except those who had been receiving a stable dose for ≥ 4 weeks prior to the study.

### Randomization

The patients were randomly allocated to either the propofol group (Group P) or propofol-remimazolam group (Group PR) at a 1:1 ratio by an investigator blinded to the study using a computer-based random number sequence generator and the sealed envelope method. Propofol was replaced with remimazolam approximately 1 h before the end of surgery in Group P. Given the distinct properties of the two anesthetics, it was not feasible to blind the anesthesiologist to the group allocation. Therefore, only the patients, parents, and study investigators were unaware of the group allocation.

### Anesthesia and perioperative management

All patients received standardized anesthetic care. Patients entered the operating room without premedication and underwent routine monitoring, including electrocardiography, pulse oximetry, non-invasive blood pressure, thermometry (3 M™ Bair Hugger™ Temperature Monitoring Patient Sensor, USA), acceleromyography (ToF scan®, Idmed, France), and patient state index (PSI, SedLine®, Masimo, USA).

In both groups, general anesthesia was induced by administering propofol through target-controlled infusion (TCI) (Injectomat TIVA Agilia® system, Fresenius Kabi, Germany) at an effect site concentration of 3.0–5.0 µg mL^− 1^. Following the confirmation of loss of consciousness, 0.6 mg kg^− 1^ of rocuronium and 3.0–5.0 ng mL^− 1^ Ce of remifentanil were administered. For propofol and remifentanil, the Schneider and Minto models were used as the pharmacokinetic models, respectively. Nasotracheal intubation was performed using a video laryngoscope (C-MAC® video laryngoscope; Karl Storz, Germany) after confirming sufficient muscle relaxation. Rocuronium was administered at an induction dose of 0.6 mg kg^− 1^ and a maintenance dose of 0.15 mg kg^− 1^ was administered to maintain a moderate neuromuscular block if the train of four (TOF) count was 4 or if spontaneous respiration occurred.

In Group P, anesthesia was maintained by adjusting the amount of propofol (1.5–5 µg mL^− 1^ Ce) using TCI while maintaining a PSI range of 25–50 until the end of surgery.

In Group PR, anesthesia induction and maintenance were performed in the same manner as in Group P, with propofol being replaced with remimazolam 1 h before the end of surgery; furthermore, the remimazolam maintenance dose was adjusted to a continuous infusion rate of 1–2 mg kg^− 1^ h^− 1^ at a PSI range of 25–50.

Remifentanil was administered via TCI in both groups, with the infusion rate being adjusted to 0.1–4 ng mL^− 1^ depending on the patient’s hemodynamic status. If the mean arterial blood pressure of the patient was < 65 mmHg despite being controlled with remifentanil, it was corrected via the administration of ephedrine or phenylephrine. All patients received 30 mg of ketorolac, 5 mg of dexamethasone, and 0.075 mg of palonosetron 1 h before the end of surgery. If the patient had a contraindication to ketorolac, 1 g of paracetamol was administered. Continuous infusion of general anesthetics was discontinued at the end of surgery, and sugammadex was administered at a dose of 2–4 mg kg^− 1^ to reverse neuromuscular blockade. Sugammadex was administered at a dose of 2 mg kg^− 1^ in patients with a moderate level of neuromuscular block indicated by a TOF count of 2 and 4 mg kg^− 1^ in those with a deep neuromuscular block indicated by a post-tetanic count of 1–2.

In Group PR, 0.5 mg of flumazenil was administered after neuromuscular blockade reversal. Tracheal extubation was performed after adequate spontaneous respiration, recovery of the airway reflex, and eye-opening, and the patients were transferred to the PACU.

In the PACU, 1 g of paracetamol was administered for pain relief if the numerical rating scale or Wong–Baker Faces pain scale score exceeded 6. However, if paracetamol had been intraoperatively administered due to contraindications to ketorolac, 20 mg of nefopam mixed in 100 mL normal saline was administered intravenously over a period of 30 min. If the patient experienced PONV, 10 mg of metoclopramide was administered intravenously as a rescue antiemetic. Patients were discharged from the hospital if they scored ≥ 9 on the Post Anesthetic Discharge Scoring System [21] or, as appropriate, according to the judgment of an anesthesiologist blinded to the group allocation.

### Outcome assessment

Data regarding age, sex, height, weight, BMI, ASA physical status classification, underlying medical conditions, current medications, duration of anesthesia, duration of surgery, and total amount of anesthetic drugs were collected from the medical records of each patient. Furthermore, the Korean version of the Quality of Recovery-15 questionnaire (QoR-15 K) was administered to the patient’s parent or legal guardian 24 h after the patient was discharged from the PACU via telephone by an investigator blinded to the group allocation [22, 23]. We also checked for other complications and re-sedation. The primary study outcome was the duration of PACU stay, which was determined as the time from when a patient entered the PACU to when they met the appropriate discharge criteria. The secondary outcomes included the time between the end of general anesthesia and initial eye-opening, the time of extubation, the initial modified Aldrete score recorded in the PACU, the occurrence of PONV, and the QoR-15 K score at 24 h. Moreover, the use of intraoperative vasopressors, PSI values at the end of surgery, and the use of rescue analgesics and antiemetics in the PACU were also investigated. In addition, the mean arterial blood pressure and PSI were retrospectively obtained from electronic medical records. The stability of the intraoperative hemodynamic profile and anesthetic depths was compared based on the median performance error (MDPE, %), median absolute performance error (MADPE, %), and wobble (%) between the groups as well as before and after replacing propofol with remimazolam in Group PR [24]. Performance measurement (PM) is a quantitative method developed for use in pharmacokinetic studies to assess the difference between the measured and predicted concentrations of a drug [25]. The most frequently used PM variables are MDPE, MADPE, and wobble, which are used to measure bias, accuracy, and time-dependent variation in repeatedly measured values, respectively. In clinical practice, these variables can be used to evaluate hemodynamic instability by measuring significant deviations in blood pressure from the reference value [24–26]. A negative MDPE is indicative of relative hypotension, whereas a substantial wobble indicates unstable blood pressure characterized by fluctuations above or below the mean arterial blood pressure. The reference value for mean arterial blood pressure was based on blood pressure measured in a quiet place with a parent or legal guardian present prior to admission to the operating room. For uncooperative patients, blood pressure was determined based on blood pressure measured at the pre-anesthesia evaluation outpatient clinic. The reference value for the PSI was set as 38, based on the range recommended for general anesthesia.

### Statistical analysis

In our preliminary study, the duration of PACU stay in Group PR was reduced by 13% compared with that in Group P, and the calculated effect size was 0.92. To achieve a power of 80% and a significance level of 5%, 21 patients had to be included in each group. Considering a dropout rate of 20%, 54 patients had to be included (27 patients per group).

Categorical variables were analyzed using the χ^2^ test or Fisher’s exact test and are presented as frequencies or numbers (percentages). Continuous variables were analyzed using Student’s *t*-test or the Mann–Whitney U test and are presented as mean ± standard deviation or median (interquartile range). A paired t-test or Wilcoxon signed-rank test was performed for within-group comparisons. The Shapiro–Wilk test was performed to evaluate the normality of data distribution. Statistical analyses were conducted using *jamovi* software, version 2.3.26 (The jamovi project, Sydney, Australia), and *R* software, version 4.3.0 (R Foundation for Statistical Computing, Vienna, Austria). *P*-values of < 0.05 were considered statistically significant.

## Results

**Fig. 1:**
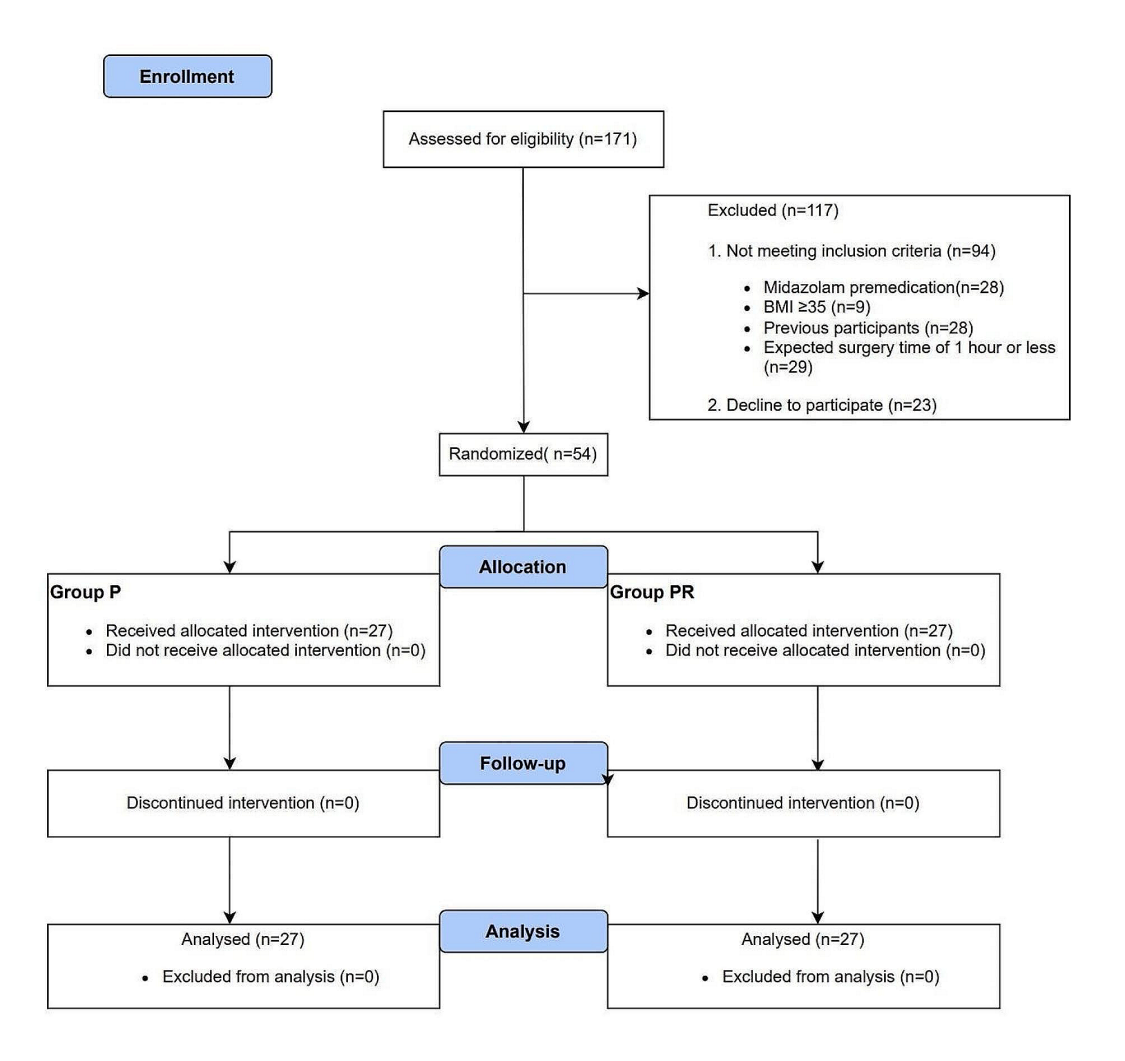
Consort diagram of patient enrollment

**Fig. 2:**
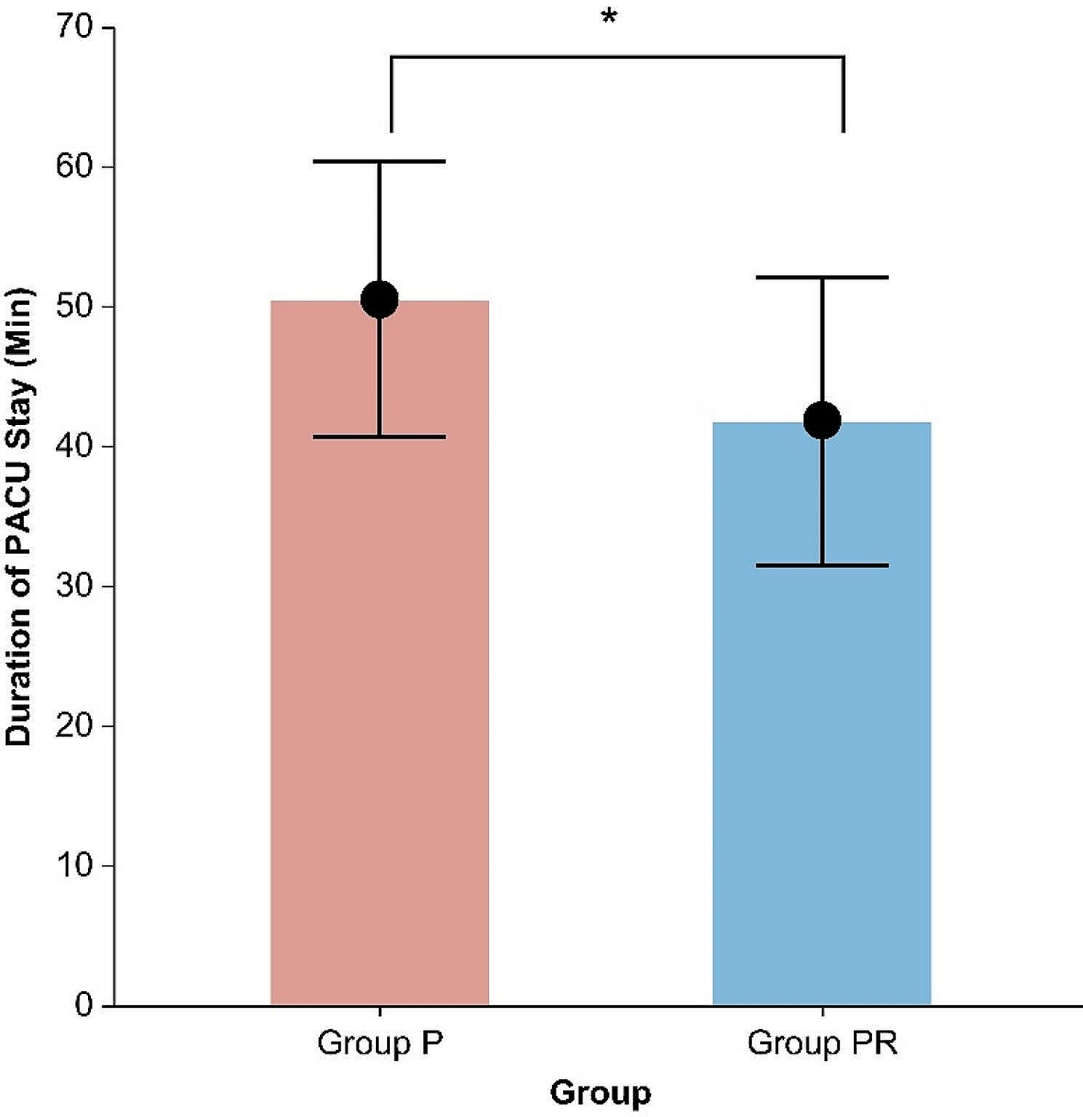
Comparison of PACU stay between Group P and Group PR. The round symbols represent the mean, whereas the upper and lower whiskers represent the standard deviation. *P = 0.002. PACU, post-anesthesia care unit; Group P, propofol group; Group PR, propofol-remimazolam group

Between December 2022 and May 2023, 171 patients were assessed to determine their eligibility for inclusion in the study; among them, 54 were enrolled and randomized into Group P (*n* = 27) or Group PR (*n* = 27). No patients were excluded after enrollment, and there were no missing data. Thus, data from 54 patients were analyzed (Fig. 1). Table 1 summarizes the patient characteristics and intraoperative data, which were well balanced between the groups, except for variations in height and weight. Regarding the primary outcome, the duration of PACU stay was significantly shorter in Group PR than in Group P (41.9 ± 10.3 min vs. 50.6 ± 9.8 min, *P =* 0.002), with a mean difference of 8.7 min (95% confidence interval [CI], 3.3–14.2) (Fig. 2). As shown in Table 2, the time to eye-opening was significantly shorter in Group PR than in Group P (2.9 [1.6–5.0] min vs. 8.3 [5.9–12.6] min, *P <* 0.001), with a median difference of 5.4 min (95% CI, 3.3–8.1). Furthermore, the time to extubation was significantly shorter in Group PR than in Group P (4.8 [2.9–6.7] min vs. 11.2 [7.8–14.4] min, *P* < 0.001), with a median difference of 5.5 min (95% CI, 3.6–7.9).

**Table 1:** Patient characteristics and intraoperative data

	Group P (*n* = 27)	Group PR (*n* = 27)	*P*-value
Age (years)	31 [27–42]	33 [23–38]	0.808
Sex (male/female), n (%)	11 (40.7)/16 (59.3)	15 (55.6)/12 (44.4)	0.276
Height (cm)	157.2 ± 10.9	163.1 ± 9.2	0.035
Weight (kg)	55.0 ± 16.4	64.3 ± 16.7	0.047
BMI (kg/m^2^)	22.0 ± 5.3	23.9 ± 4.9	0.199
ASA physical status (II/III), n (%)	4 (14.8)/23 (85.2)	6 (22.2)/21 (77.8)	0.484
Use of antiepileptic drugs, n (%)	13 (48.1)	17 (63)	0.273
Preoperative QoR-15 K score	103.2 ± 15.0	109.6 ± 15.3	0.128
Duration of surgery (min)	150 [98–225]	180 [113–215]	0.359
Duration of anesthesia (min)	175 [114–243]	190 [128–228]	0.50
Duration of remimazolam infusion (min)		64.7 ± 25.0	
Total amount of propofol (mg)	950 [700–1550]	800 [500–1090]	
Total amount of remimazolam (mg)		60.0 [32.5–70.0]	
Total amount of remifentanil (mcg)/Weight (kg)	10.6 [6.70–13.1]	10.7 [8.37–14.3]	0.268
Need for vasopressors, n (%)	5 (18.5)	4 (14.8)	0.715

Values are presented as mean ± SD, median [interquartile range], or number of patients (%)

Group P: propofol group; Group PR: propofol-remimazolam group; BMI: body mass index; ASA: American Society of Anesthesiologist; QoR-15 K: Korean version of the Quality of Recovery-15

**Table 2:** Comparison of emergence variables and postoperative outcomes between Groups P and PR

	Group P (*n* = 27)	Group PR (*n* = 27)	Mean or Median difference (95% CI)	*P*- value
Time to eye-opening (min)	8.3 [5.9–12.6]	2.9 [1.6–5.0]	5.4 (3.3–8.1)	< 0.001
Time to extubation (min)	11.2 [7.8–14.4]	4.8 [2.9–6.7]	5.5 (3.6–7.9)	< 0.001
Modified Aldrete Score	8 [8–8]	8 [7–9]	0 (0–0)	0.933
Need of rescue analgesics at PACU	0	0		
PONV at PACU	0	0		
Postoperative QoR-15 K score	108.8 ± 13.4	112.6 ± 13.5	-3.8 (-11.1 to 3.6)	0.306

Values are presented as mean ± SD or median [interquartile range]

Group P, propofol group; Group PR, propofol-remimazolam group; PONV, postoperative nausea and vomiting; PACU, post-anesthesia care unit; QoR-15 K: Korean version of the Quality of Recovery-15

None of the patients required rescue analgesics in the PACU, and the incidence of PONV was not reported. There were no significant between-group differences in the modified Aldrete and postoperative QoR-15 K scores (Table 2). Further, there were no significant between-group differences in the intraoperative hemodynamic and anesthetic depth stability based on PMs (Table 3). However, the mean arterial pressure MDPE (%) was significantly lower in Group P than in Group PR (-18.5 ± 11.5 vs. -10.7 ± 11.2, *P =* 0.021). In Group PR, no significant differences in the hemodynamic and anesthetic depth stability were observed before and after replacing propofol with remimazolam (Table 4). The PSI values were maintained within the 25–50 range after conversion to remimazolam in all but four patients. These four patients experienced a transient reduction in PSI values to ≤ 25, which subsequently returned to baseline levels within 15 min.

**Table 3:** Comparison of the intraoperative performance measurement between the groups

	Group P (*n* = 27)	Group PR (*n* = 27)	Mean or Median difference (95% CI)	*P* -value
Intraoperative mean arterial blood pressure				
MDPE, %	-18 ± 11.5	-10.7 ± 11.2	-7.4 (-13.6 to -1.2)	0.021
MDAPE, %	19.4 ± 9.6	12.9 ± 8.8	6.5 (1.5–11.5)	0.013
Wobble, %	4.0 [2.9 − 5.2]	4.1 [3.2 − 5.5]	-0.2 (-1.2 to 0.7)	0.528
Intraoperative PSI value				
MDPE, %	-7.2 ± 19.1	-14.3 ± 16.7	7.1 (-2.7 to 16.9)	0.154
MDAPE, %	21.3 ± 10.7	22.7 ± 9.9	-1.4 (-2.7 to 16.9)	0.624
Wobble, %	10.7 [8.0 − 16.7]	10.7 [7.3 − 16.0]	0 (-1.3 to 4.0)	0.489
PSI at the end of surgery	45 [33 − 51]	42 [34 − 55]	-2 (-10 to 7)	0.993

Values are presented as mean ± SD or median [interquartile range]

Group P: propofol group; Group PR: propofol-remimazolam group; MDPE: median performance error; MDAPE: median absolute performance error; PSI: patient state index; CI: confidence interval

**Table 4:** Comparison of the intraoperative performance measurement between propofol-based and remimazolam-based TIVA (Group PR)

	Group PR (*n* = 27)		Mean or Median difference (95% CI)	*P*-value
	Propofol-based TIVA	Remimazolam-based TIVA		
Intraoperative mean arterial blood pressure				
MDPE, %	-9.8 ± 11.1	-11.1 ± 11.8	1.3 (-0.8 to 3.3)	0.230
MDAPE, %	12.2 ± 8.6	13.8 ± 8.8	-1.5 (-3.3 to 0.2)	0.082
Wobble, %	3.9[3.0–5.8]	3.8[2.7–4.2]	0.9 (-0.3 to 2.2)	0.104
Intraoperative PSI value				
MDPE, %	-17.3 ± 18.4	-10.8 ± 18.6	-6.6 (-16.7 to 3.6)	0.632
MDAPE, %	22.9 ± 12.2	23.2 ± 10.9	-0.2 (-6.6 to 6.1)	0.575
Wobble, %	8.0 [4.0–10.7]	10.7 [5.3–16.0]	-3.2 (-7.2 to 0.9)	0.123

Values are presented as mean ± SD or median [IQR]

Group PR: propofol-remimazolam group; MDPE: median performance error; MDAPE: median absolute performance error; PSI: patient state index; CI: confidence interval

## Discussion

This study explored the effects of replacing propofol with remifentanil 1 h before the completion of dental treatment, followed by flumazenil reversal. The results indicated that this approach led to faster recovery and shorter PACU stay than propofol-based TIVA alone.

Previous studies comparing the duration of PACU stay between propofol-based TIVA and remimazolam-based TIVA with the additional use of flumazenil have reported inconsistent findings. A previous study reported no significant difference in the duration of PACU stay [17], whereas another study reported a significantly shorter PACU stay using the latter approach [19]. The first study included patients with ASA I–II who underwent open thyroidectomy and received 0.2 mg of flumazenil, with patients who achieved a modified Aldrete score of ≥ 9 being discharged to the ward [17], whereas the other study included patients with ASA II–III who received 0.5 mg of flumazenil [19].

The administration of flumazenil (0.5 mg) following remimazolam has demonstrated efficacy in facilitating recovery from anesthesia or sedation [15, 19, 27, 28]. Although benzodiazepine premedication was not included in the present study, it is frequently required in patients with mental disabilities due to poor cooperation during anesthesia induction. In such cases, the administration of flumazenil may prove beneficial in facilitating recovery. However, caution must be exercised given the potential for re-sedation approximately 1–2 h postoperatively and the lack of evidence supporting the beneficial effects of excessive administration of flumazenil [29, 30]. In the current study, the patients recovered in the PACU and remained in the hospital for at least 2 h postoperatively. Although our study, did not include a case of re-sedation, it is imperative to remain vigilant for the occurrence of re-sedation.

The incidence of PONV was not reported in our study, suggesting that replacing propofol with remimazolam does not increase the incidence of PONV in our study. Propofol is known for its ability to prevent PONV, even when administered in small doses [31–33]. A recent study demonstrated that a small dose of propofol and dexamethasone in remimazolam-based TIVA effectively prevented PONV [34]. This was also evident in our empirical findings. In addition, we administered palonosetron to prevent PONV.

We observed no significant between-group differences in the postoperative QoR-15 K scores. Previous studies have compared postoperative QoR scores between propofol-based TIVA and remimazolam-based TIVA. Some studies have reported no significant between-group differences [16, 17], whereas one study reported a decrease in postoperative QoR scores in the remimazolam-based TIVA group [35]. Cessation of remimazolam administration may lead to undesirable desensitization effects and the incidence of rebound phenomena such as anxiety [13]. These phenomena may contribute to the decreased postoperative QoR scores in remimazolam-based TIVA [35]. However, none of the patients in Group PR showed a rebound phenomenon in our study.

Compared with propofol-based TIVA, remimazolam-based TIVA is associated with a relatively low incidence of hypotension [14, 19, 36]. Our findings indicated lower MDPE in Group P than that in Group PR. Nevertheless, this was within the normotensive range, and there was no significant between-group difference in the requirement for vasopressors. In addition, there was no significant between-group difference in intraoperative hemodynamic and anesthetic depth stability based on PMs, even after the replacement of propofol with remimazolam in Group PR.

Previous studies have demonstrated that remimazolam-based TIVA can lead to elevated PSI values compared with propofol-based TIVA, with some values exceeding the threshold of 50 [16, 37]. Additionally, there have been reports of chronic benzodiazepine users developing tolerance to remimazolam, necessitating the use of alternative anesthetics or higher remimazolam dosages [38, 39]. Although our study included chronic benzodiazepine users, we did not observe any such discrepancies. Several studies have reported that both remimazolam and propofol act on GABA-A receptors, leading to a synergistic effect [40, 41]. This effect may explain the presence of four cases in our study who experienced a transient reduction in PSI values to ≤ 25 after changing to remimazolam and why chronic benzodiazepine users included in our study did not show remimazolam tolerance and maintained their PSI effectively.

This study has several limitations. First, this study was performed at single center and had a limited sample size. Second, the assessment of QoR scores relied on responses from parents or legal guardians, which may have impeded accurate assessment of the patients’ recovery experiences. Third, the timing of anesthetic conversion to remimazolam varied among patients given the challenge of accurately predicting the duration of the surgical procedure. Lastly, our study focused on patients with mental disabilities, who often have comorbidities and receive various medications. Future research is required to validate the findings of this study.

## Conclusions

In the context of outpatient general anesthesia for dental treatment in patients with mental disabilities, replacing propofol with remimazolam 1 h before the end of dental treatment and reversal with flumazenil improved recovery rates and reduced the duration of stay in the PACU, without the incidence of any adverse effects. Thus, this protocol can be considered a safe and effective anesthetic approach that prioritizes both patient safety and efficiency.

## Data Availability

The datasets used and/or analysed during the current study are available from the corresponding author on reasonable request.

## Abbreviations

PONV: Post postoperative nausea and vomiting
ASA: American Society of Anesthesiologists
TIVA: Total intravenous anesthesia
PACU: Post-anesthesia care unit
BMI: Body mass index
Group P: Propofol group
Group PR: Propofol-remimazolam group with flumazenil reversal
PSI: Patient state index
TCI: Target-controlled infusion
QoR-15 K: Korean version of the Quality of Recovery-15 questionnaire
TOF: Train of Four
MDPE: Median performance error
MADPE: Median absolute performance error
PM: Performance measurement
CI: Confidence interval
SD: Standard deviation
IQR: Interquartile range
